# Influential factors on learning through the hidden curriculum in the perspective of undergraduate baccalaureate nursing students

**Published:** 2014-04

**Authors:** ZOHREH KARIMI, TAHEREH ASHKTORAB, EESA MOHAMMADI, HEIDARALI ABEDI

**Affiliations:** 1 Department of Nursing, Nursing & Midwifery School, Ahvaz Jundishapur University of Medical Sciences, Ahvaz, Iran;; 2 Department of Nursing, Nursing & Midwifery School, Shahid Beheshti University of Medical Sciences, Tehran, Iran;; 3 Department of Nursing, Medical Sciences School, Tarbiat Modares University, Tehran, Iran;; 4 Department of Nursing, Nursing & Midwifery School, Khorasgan (Isfahan) Branch, Islamic Azad University, Isfahan, Iran

**Keywords:** Curriculum, Influencing factors, Education, Students

## Abstract

**Introduction**: Nursing curriculum is not always overt; it can also exist covertly in the form of a hidden curriculum. This study aims to explain the factors influencing learning through the hidden curriculum in the perspective of undergraduate baccalaureate nursing students.

**Methods**: This qualitative study was conducted through purposeful sampling strategy on 24 undergraduate baccalaureate nursing students studying in the first to the fourth years of their education. The data were collected using semi-structured interviews and this process continued until data saturation and categories’ emergence. Inductive content analysis was used for data analysis.

**Results**: Professional promotion as a learning factor, impact of personal characteristics on learning, educator’s behavior as a learning stimulus, and feedback as a learning stimulus are the main categories emerged in this study; some of them included sub-categories as well.

**Conclusion**: Professional promotion, personal characteristics, educator’s behavior and feedback were the main influencing factors on learning through the hidden curriculum in undergraduate baccalaureate nursing students. The findings of this study can be used for developing strategies to promote nursing education and as a result patient care. Further studies are recommended to identify other factors.

## Introduction

Nursing is the largest health care profession (1) and plays an essential role in patient care; however, the nature and the quality of performing this role depend on preparation of the individual for such performance and, to put it simpler, on the nursing education, which is often administered within a four-year academic period. The purpose of nursing education is to encourage the nursing students to acquire knowledge, practical skills and social responsibility required for accepting their roles as professional nurses upon accomplishment of the nursing program (2).

Nursing curriculum is defined as the totality of the philosophical approaches, curriculum outcome statements, overall design, courses, teaching-learning strategies, delivery methods, interactions, learning climate, evaluation methods, curriculum policies, and resources (3). A curriculum is more than just a syllabus or a statement of content; it is about what should happen in a teaching program, as well as about the intention of the teachers and the manner they manage this to happen (4). This is not always overt and can also exist covertly within a hidden curriculum (5). The account of the curriculum content is not complete unless the concept of the hidden curriculum is taken into consideration as well (4).

The influences of the hidden curriculum function at organizational structure and culture level (6). The hidden curriculum covers a set of unwritten social and cultural values, rules, assumptions and expectations, and has much stronger effects on the individuals’ behavior as compared to the formal curriculum (7). Learning through the former is a more powerful experience for students than through the latter, and also the messages transmitted by the hidden curriculum are mainly influenced by culture. The inconsistency between the formal and the hidden curricula challenges the students with complicated emotional and ethical problems (8).

Furthermore, since learning through this implicit curriculum is experienced in real-life social settings and not in the classroom, it is pervasive, continually reinforced, and naturally accepted by faculty and trainees as “how things work”. The hidden curriculum is generally defined as the values, beliefs, and expectations of institutional and professional culture that shape the students' learning (9). In the health sciences, including nursing, the most common realms regarding the hidden curriculum deal with professionalism, role modeling, leadership, ethics, and the ways of thinking and beginning a professional (10). The hidden curriculum conveys positive and negative messages (11).

There are reportedly more deep social bonds among Asian students as compared to Western countries; hence, the higher influence of hidden curriculum on these students than on their western counterparts is expected (12). In Iran, students can study nursing across all higher education levels from bachelor to doctoral (13). In nursing, there is a four year baccalaureate program accredited by the High Council of Medical Education of the Ministry of Health and Medical Education (14). This is the basic nursing program at the academic level, aiming to train competent nurses with the necessary theoretical knowledge and technical skills in order to provide high quality general nursing care (13).

Much research has so far been conducted on the factors affective on learning; however, there are few documented studies reporting the influencing factors on learning through the hidden curriculum. Qualitative studies are useful for investigating the cultural and contextual aspects of education (9); they are also quite appropriate to answer questions about how learners and teachers make sense of the educational events in which they participate, complex learning environments, subtle learning relationships; learning outcomes that are best described rather than counted or measured, and previously unexplored topics in medical education (15). Therefore, this qualitative study was carried out to explain the factors affecting learning through the hidden curriculum in Iranian undergraduate baccalaureate nursing students.

## Methods

In this qualitative content analysis study, the participants were selected through purposeful sampling strategies, and the sampling continued until data saturation. To do this, 24 nursing students from the School of Nursing and Midwifery (Ahvaz Jundishapur University of Medical Sciences) were deeply interviewed in 2012. The students were in the first and fourth years of baccalaureate nursing program; they were also volunteers and willingly participated in the interview. Data were collected through semi-structured and face-to-face interviews. The interviews started with this general question: "Please tell us about your experiences of learning  materials beyond the formal curriculum of the university” and then  the responses of the participants guided us to ask probing questions such as "How have you learned these things?" and "What factors have led you to learn these things?"  The interviews lasted about 60 to 90 minutes. The interviews were recorded and then, at the first opportunity, they were listened accurately several times and transcribed verbatim.

Data analysis began with data collection simultaneously. Inductive content analysis was used for data analysis. In this research, the content of each interview formed an analysis unit. After reading the interview texts several times, meaning units were specified, and after condensation and abstraction, they were labeled with the appropriate code. The codes were reviewed several times so that they were categorized based on semantic similarity into certain sub-categories and categories.

To ensure data credibility, the researcher carried out a prolonged engagement for 12 months for sampling and data analysis simultaneously. We used comments and views of colleagues to approve and modify the extracted codes and categories, if needed. The extracted codes were returned to the participants for approval. The expert review was used as well.

**Ethical Considerations**

At the beginning, the study was approved by the Ethics Committee of Ahvaz Jundishapur University of Medical Sciences, and formal permissions were obtained from the Nursing and Midwifery School to access the participants. All participants in the study were informed of the research purposes, and the interviews were recorded based on their consent. Meanwhile, they were assured that all of their information will remain confidential, and that the audio files will be deleted after use.

## Results

The research participants were 24 undergraduate baccalaureate nursing students (67% female and 33% male) with the age range of 21-26 years, who were studying in their first to the fourth years of their education.

Data analysis consisted of 4 main categories including; 1) professional promotion as a learning factor, 2) influence of personal characteristics on learning, 3) educator’s behavior as a learning stimulus, and 4) feedback as a learning stimulus. Some of the above categories consisted sub-categories (Table 1).

**Table 1:** Categories and sub-categories of influencing factors on learning through the hidden curriculum

**Categories**	**Sub-categories**
Professional promotion as a learning factor	- Patient centeredness as a learning stimulus - Professional autonomy as a learning stimulus - Improving the professional image as a learning stimulus
Influence of personal characteristics on learning	- Personal needs as a learning stimulus - Values as a learning stimulus
Educator’s behavior as a learning stimulus	-
Feedback as a learning stimulus	-

1. **Professional promotion as a learning factor**
  One of the main categories extracted was professional promotion as a learning factor, with the sub-categories including "patient-centeredness as a learning stimulus", "professional autonomy as a learning stimulus", and "improving the profession image as a learning stimulus".
  1. **Patient-centeredness as a learning stimulus**
    The students mentioned factors such as paying attention to patient, and deficiencies in patient care as the stimuli to learn through the hidden curriculum:
*“The main thing that encouraged me to learn things beyond the formal curriculum is the patient’s life and his/her needs”* **(Participant 1).**
*“When I entered the clinical setting, I saw patients that we could do many things for them but nobody cared about them; for example, having true communication with patient”* **(Participant 10).**
  2. **Professional autonomy as a learning stimulus**
    This category indicates that students were trying to play an independent role and they could cope with the tasks they were expected to perform in future. In this regard, a participant stated:
*“I am not supposed to be a student forever and always need an educator to control me. I am supposed to be independent in future to become a nurse, not a trainee; a nurse who is trustworthy to the patient and because of this, he/she has come to this hospital”* **(Participant 19).**
*“As a trainee, we used to go to different wards in different hospitals; there I saw behaviors that attracted my attention. I felt if I learned them, they would be useful, and they can be necessary in my future career”* **(Participant 24).**
  3. ** Improving the professional image as a learning stimulus**
    Positive attitude of society as a factor of effort to improve abilities and becoming a better nurse were of the main concerns of the students in the present study as the learning stimuli. Examples of student statements are listed below:
*“All I learned apart from the formal curriculum is useful both for me and for my patients. The patient will have a positive attitude toward nurses, and when he/she recovers and returns to the society, he/she will tell others: ‘The nurse had a good communication with me’; this is important to me, and by this, the community’s attitude toward male nurses will change into more positive  step by step”* **(Participant 8).**
*“To be a good nurse was main concern, so I willingly try to learn anything that may help me in the future”* **(Participant 5).**
2. **Influence of personal characteristics on learning**
  One of the other main categories extracted in this work was influence of personal characteristics on learning, with the sub-categories of personal needs as a learning stimulus, and values as a learning stimulus.
  1. **Personal need as a learning stimulus**
    This sub-category shows that different needs of students are effective factors in their learning through the hidden curriculum:
*“Sometimes, a scientific need makes you go to the library or a person; in addition to that scientific topic, you can learn more others things over there”* **(Participant 1).**
*“As a student, an educated person and a nurse, there is a need to learn having true and correct communications with patients and colleagues and also doing things in a correct way”* **(Participant 12).**
* “Everybody needs others’ attention; when I’m responsible and care about my patient, this will make me achieve anything I want to gain, that is being seen by others”* **(Participant 7).**
  2. **Values as a learning stimulus**
    This sub-category indicates that the student tries to learn materials beyond the formal curriculum because values are important to families. Teaching values start from families and will be developed in other contexts. One of the students said:
*“When I was in the first year of my baccalaureate nursing education and I wanted to enter the hospital, my mother told me: ‘About your communication with patients, you have to respect them and do not be arrogant’. In my family, subjects like job conscience, honesty and respect for others are values. When these are values for you, you will go for them and learn them. But if they are not values for you, you won’t care about them”* **(Participant 11).**
3. **Educator’s behavior as a learning stimulus**
  Different behaviors of educators can teach values to students and make them interested in them:
*“In his communication with me, the educator created a sense of altruism in me, and now I can do the same for my patients and colleagues”* **(Participant 2).**
* “My interest in nursing resulted in learning more things, or learning these items became important for me.  At first, it started with my interest in this major; however, this was my educator’s behavior that made me interested in it”* **(Participant 13).**
4. **Feedback as a learning stimulus**
  This category reflects that others’ behavior is a feedback to students and is an effective factor in learning through the hidden curriculum:
*“Others’ interaction with me was an effective factor to learn about communication with patients or educators”* **(Participant 3).**
* “One of the factors, which caused me to learn new items beyond the formal curriculum was when I did something positive for my patient, there was his/her feedback and he/she thanked and appreciated me”* **(Participant 11).**

## Discussion

The experiences of the students participating in this study show that there are factors influential on learning through the hidden curriculum. “Professional promotion as a learning stimulus”, “influence of personal characteristics on learning”, “educator’s behavior as a learning stimulus”, and “feedback as a learning stimulus” were the categories obtained in the analysis of the data related to the participants’ experiences.

One of the main categories was “professional promotion as a learning factor” with the subcategories such as patient-centeredness, professional autonomy, and improving the professional image. 

The results showed that patient-centeredness was a stimulus for nursing students to learn through the hidden curriculum, implying that patient-centeredness may be developed in academic context (16).

“Professional autonomy” was another learning factor in the perspective of the nursing students in the present study. Autonomy is the keystone of every profession. It is clear that graduate students with low levels of autonomy will not be able to demonstrate their professional attitudes and behaviors effectively. Sine these nursing students are going to provide individuals, families and society with health care in future, they have to be brought up as individuals enjoying high levels of autonomy in order to improve public health and quality of life, comfortably communicate in society, and use professional knowledge and skills to make more powerful decisions. Some aspects of autonomy deficiency are closely related to education. Unfortunately, the ongoing nursing curriculum programs are designed and implemented in such a way that future professional nurses are not adequately able to improve the students’ thought and acting like independent professionals; some may even decrease the students’ levels of autonomy (17).

“Improving the professional image” was among the other factors mentioned by the participants in the present study as a motivating factor of learning through the hidden curriculum. It is necessary to improve the image of nurses and also the nursing profession in the society in order to attract more students to this profession (18). Creating a more professional image for nurses can increase their power in many aspects including the clinical setting, the research, university circles, the society and government (19).

The category of “influence of personal characteristics on learning” included two sub-categories:  personal needs as a learning stimulus, and values as a learning stimulus. The findings suggest that maintaining personal values, and encouraging compassion and communication were important to the student nurses so that they expressed surprise and disillusionment when their expectations of the profession did not match with the reality (20).

“Values as a learning stimulus” was another factor of the effect of personal characteristics on learning through the hidden curriculum in the present work. There is little information on how the values are brought upon admission to the discipline or how the professional values are actually being assimilated by graduation (21).

“Educator’s behavior” was mentioned to be one of the influencing factors on learning through the hidden curriculum. It has been suggested that, considering knowledge and the values concerning educational roles and responsibilities, professional behaviors in teaching and scholastic relationships cannot change quickly. Learners observe closely what the educators and other educational team do and how they behave in educational and health centers. On the other hand, they actively follow how the educators and others think, say, or interact with the students or with other clients on a daily basis (22). Such influences can be experienced from the first contacts of the educators with students (23).

Another factor influencing learning through the hidden curriculum was “feedback”, which is an essential element of both teaching and learning that connects education and assessment. Hence, feedback deals with strengthening and reiterating admirable behavior, and also modifying and improving the erroneous and wrong ones (24).

## Conclusion

As a conclusion, in the present work, professional promotion (patient centeredness, professional autonomy and improving the professional image), personal characteristics (personal needs and values), educator’s behavior, and feedback were found to influence learning through the hidden curriculum in the baccalaureate undergraduate nursing students. Also the results of this research can be used for developing strategies to promote nursing education and patient care. Conducting more research for identification of other possible factors is recommended. Generalization of the results of this study should be made with caution because of the limitations inherent in all qualitative studies such as this. 
